# The predictive value of CTSI scoring system in non-operative management of patients with splenic blunt trauma: The experience of a level 1 trauma center

**DOI:** 10.1016/j.ejro.2023.100525

**Published:** 2023-09-23

**Authors:** Ali Barah, Ayman Elmagdoub, Loai Aker, Yaman M. Alahmad, Zeyad Jaleel, Zahoor Ahmed, Rahil Kaassamali, Ammar Al Hasani, Hassan Al-Thani, Ahmed Omar

**Affiliations:** a Radiology Department, Hamad Medical Corporation, Doha, Qatar; b Trauma Department, Hamad Medical Corporation, Doha, Qatar

**Keywords:** Splenic injury, Interventional radiology, Embolization, CT severity index, Trauma

## Abstract

**Background:**

The spleen is one of the most injured organs following blunt abdominal trauma. The management options can be either operative or non-operative management (NOM) with either conservative management or splenic artery embolization. The implementation of CT in emergency departments allowed the use of CT imaging as a primary screening tool in early decision-making. Consecutively, new splenic injury scoring systems, such as the CT severity index (CTSI) reported was established.

**Aim:**

The main aim of this study is to evaluate the effect of the implementation of CTSI scoring system on the management decision and outcomes in patients with blunt splenic trauma over 8 years in a level 1 trauma center.

**Methods:**

This is a retrospective study including all adult patients with primary splenic trauma, having NOM and admitted to our hospital between 2013 and 2021.

**Results:**

The analyses were conducted on ninety-nine patients. The average sample age was 32.7 ± 12.3 years old. A total of (63/99) patients had splenic parenchyma injury without splenic vascular injury. There is a statistically significant association between CTSI grade 3 injury and the development of delayed splenic vascular injury (p < 0.05). There is an association between severity of initial CTSI score and the risk of NOM/clinical failure (p = 0.02).

**Conclusion:**

Our findings suggest implementing such a system in a level 1 trauma center will further improve the outcome of treatment for splenic blunt trauma. However, CTSI grade 3 is considered an increased risk of NOM failure, and further investigations are necessary to standardize its management.

## 1 Introduction

The spleen remains one of the most injured organs following blunt abdominal trauma [1], [2]. The types of splenic injury are highly variable from a subtle contusion to severe ones leading to intense intraperitoneal hemorrhage and shock. The treatment options can be either operative or non-operative management (NOM) with either conservative management (CM) or splenic artery embolization (SAE). For many years, splenectomy was advocated to control bleeding after traumatic splenic injuries [3]. Nevertheless, the immunological role of the spleen has pushed clinicians to adopt splenic salvaging NOM, which has been evolving over the past two decades. Currently, NOM is adopted as a standard method for splenic injury after blunt abdominal trauma with hemodynamic stability [3], [4], [5].

The implementation of CT in emergency departments allowed the use of CT imaging as a primary screening tool in early decision-making. This has shifted the protocols of blunt splenic trauma management in stable patients toward NOM [6], [7].

Aiming to predict the outcome and the type of management, multiple radiological scoring systems for blunt splenic injury were created based on CT imaging [8], [9]. The American Association for the Surgery of Trauma (AAST) proposed the first splenic injury scoring system in 1989 to provide a consistent descriptive guide in patient management. This scoring system was based on an anatomical description, rather than clinical pathways [8], [9], [10]. Despite the first revision of the AAST scoring system in 1994, the indications for NOM in the management of blunt splenic injury remained restrained. Indeed, vascular injuries such as contrast media extravasation, arterial pseudo-aneurysms or arteriovenous fistula have never been incorporated in the scoring system which has limited the role of NOM [3], [4], [5]. Consecutively, new splenic injury scoring systems, such as the CT severity index (CTSI) reported by Marmery et al. [11] in 2007 and the revised AAST-2018 version were developed [12]. Compared to AAST-1994, these new systems incorporate vascular abnormality providing better prediction of the patients who need NOM.

The aim of our study is to evaluate the effect of the implementation of CTSI scoring system on the management decision and outcomes in patients with blunt splenic trauma over 8 years in a level 1 trauma center.

## 2 Materials and methods

### 2.1 Study design and data collection

This is a retrospective, cross-sectional study including all adult (>16 years) patients with primary splenic trauma, having NOM and admitted to our hospital between January 2013 and December 2021. The study protocol was approved by the institutional review board (MRC-01–21–294) of the institution, waiving the requirement for informed consent. For each patient, the following data were assessed and recorded: gender, age, mechanism of injury, associated injury, clinical parameters at presentation (SpO2, heart rate, systolic blood pressure, hemoglobin level), Glasgow Coma Scale (GCS), CTSI grade, trauma intensive care unit (TICU) length stay, initial NOM management (conservative, embolization), rate of NOM success and failure, and in-hospital mortality with the cause of death.

According to the institutional protocol, blunt splenic trauma patients underwent a primary survey in the trauma unit with an assessment of response to fluid challenge. Based on Advanced Trauma Life Support (ATLS), patients would be classified into hemodynamically stable, transient responders or unstable. The first two categories underwent triphasic contrast-enhanced CT (CECT) after the primary survey with consideration for NOM [5], [10]. Patients with penetrating splenic trauma, pediatric patients, hemodynamically unstable or presenting with signs of peritonitis and patients with a history of external surgical or interventional procedures prior to initial CT assessment were excluded from this study. Also, patients with absence of triphasic CT protocol, or absence of splenic injuries at initial CECT were excluded from the study.

### 2.2 CTSI scoring system

All initial CECT were reported in emergency radiology by a consultant radiologist. The reports were then double checked retrospectively by two board certified emergency radiologists with 10 and 15 years of experience. The CECT protocol consisted of a pre-contrast scan followed by post-contrast acquisitions obtained in the arterial phase (using a bolus tracking system) and venous phase at 65 s, using a contrast volume of 2 mL/kg and injection speed of 3–4 mL/s. 3.5- to 5-mm-thick transversal and 5-mm-thick sagittal and coronal multiplanar-reformatted images were then reviewed on a picture archiving and communication system (AGFA IMPAX; AGFA Health Care). If a vascular lesion was suspected, additional image acquisition in the delayed phase was performed. The presence of splenic parenchyma injury (SPI) and the type of splenic vascular injury (SVI) were recorded based on the CECT findings. CTSI scoring system was used as the reference of NOM decisions (see Table 1). Any divergent findings on the initial CECT were jointly assessed and the results were decided by consensus before establishing the final CTSI grading. Patients with SPI such as parenchymal laceration, parenchymal hematoma, and splenic capsule disruption were divided following the severity of parenchymal injury into three groups: low-grade injury (CTSI grade 1 and 2), intermediate-grade injury (CTSI grade 3) and high-grade injury (CTSI grade 4a). In the presence of any SVI, such as pseudoaneurysm, arterio-venous fistula, contrast extravasation or abrupt vessel truncation, the score was upgraded to CTSI grade 4a. In case of the presence of peritoneal extravasation, the CTSI grade was upgraded to 4b.

**Table 1:** The Baltimore CT Severity Index (CTSI).

Grade	Description
Grade I:	- Sub capsular hematoma < 1 cm - Thick laceration < 1 cm parenchymal depth - Parenchymal hematoma < 1 cm of diameter
Grade II:	- Sub capsular hematoma 1–3 cm thick - Laceration 1–3 cm parenchymal depth - Parenchymal hematoma 1–3 cm in diameter
Grade III:	- Splenic capsular disruption - Sub capsular hematoma > 3 cm thick - Laceration > 3 cm parenchymal depth - Parenchymal hematoma > 3c in diameter
Grade IVa:	- Active intraparenchymal or sub-capsular splenic bleeding - Splenic vascular injury (extravasation, pseudo aneurysm, AV fistula) - Shattered spleen
Grade IVb:	- Active intraperitoneal bleeding

### 2.3 Non-operative managements (NOM)

**Fig. 1:**
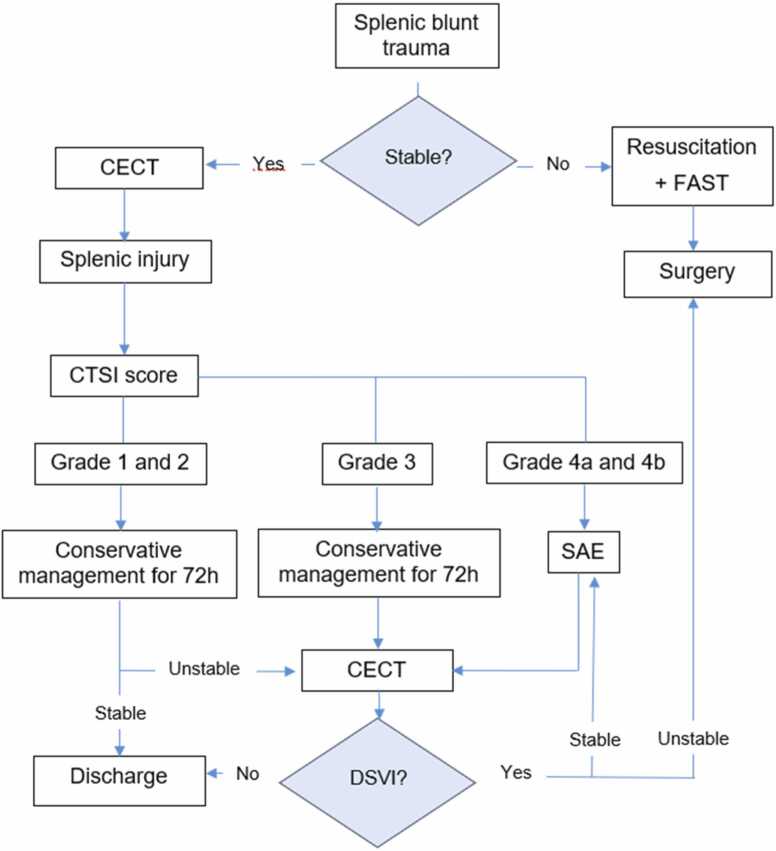
Algorithm of the management of splenic blunt trauma.

Patients with CTSI grade 1, 2 and 3 underwent CM. This consisted of bed rest with strict clinical and laboratory surveillance and observation of different vital signs every 6 h for the first 72 h. Patients with CTSI grades 4a and 4b were indicated for SAE. The procedures of SAE were performed by a team of 10 interventional radiologists, 6 consultants with 6–20 years of experience and 4 fellows with 1–3 years of experience. A posteroanterior selective arteriogram of the splenic artery was obtained using a 4Fr or 5Fr diagnostic catheter or 6 F guide catheter. Super-selective catheterization of distal splenic artery branches, when required, was performed using coaxial 2.7 F and 2.4 F microcatheters. Different SAE techniques proximal, distal, or combined and different embolic materials were used, based on the type of injury found on the CECT and angiogram. Distal embolization was defined as occlusion of a branch distal to the main splenic artery using coils 0.018 in. or gelatin sponges. It was indicated in the presence of low-grade SPI associated with a maximum of 3 focal SVIs. Proximal embolization was defined as occlusion of the main splenic artery distal to the dorsal pancreatic artery. It was indicated in case of high-grade splenic injury without SVI or in the presence of multiple SVI making individual distal embolization impracticable. Detachable or pushable coils, by using either microcatheter 2.7 F, or through the diagnostic catheter and Amplatzer Vascular Plugs (AVP) type II and IV were used based on the size of plug needed to occlude proximal splenic artery. Lastly, combined, proximal and distal embolization was performed in the presence of focal SVI and high-grade SPI using similar techniques and materials. The choice of technique of embolization and embolic material was up to the preference of the interventionalist, taking into account the number and anatomy of the injury (Fig. 1).

### 2.4 In-hospital stay and follow-up

**Fig. 2:**
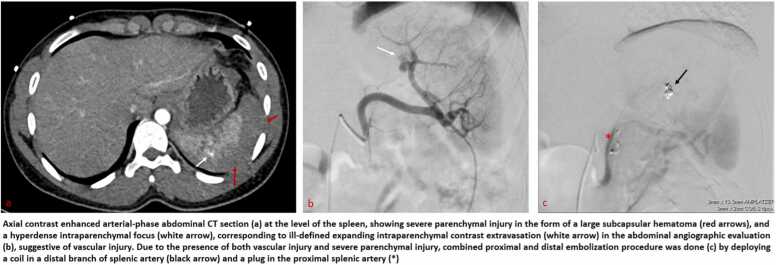
Selected images for combined embolization for a case.

Following the institutional protocol, patients with CTSI grades 1,2 or 3 were treated conservatively and discharged 72 h after having intensive clinical monitoring during their admission to the trauma unit. This stay was prolonged in case of deterioration of the clinical status requiring additional investigations during the stay. Patients with CTSI grade 3 injury and patients with grade 4a and 4b who underwent SAE had routine CECT follow-up at day three of admission. (Fig. 2). The deterioration of hemodynamic status during the admission after initial NOM were assessed by urgent CECT. If a delayed splenic vascular injury (DSVI) was detected, then patients underwent SAE or splenectomy. Patients were given appointments for followed-up after discharge, in trauma outpatient clinics for clinical evaluation. Clinical success of NOM was defined as a patient leaving the hospital with their spleen in situ. Clinical failure was defined as the need for splenectomy after an initial NOM.

**Fig. 3:**
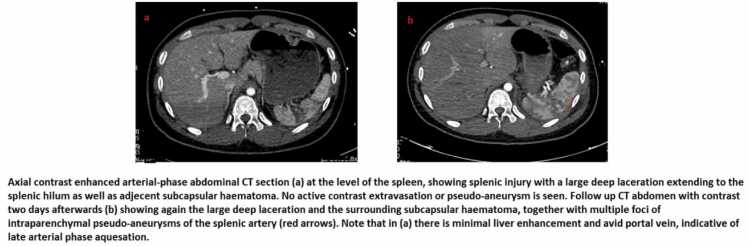
Selected images for a case of ‘’missed’’ delayed vascular injury.

DSVI was defined as having SVI on follow up CECT, that was not present on initial CECT, for patients that underwent NOM after splenic injury. Upon retrospective meticulous review of the initial CT, further classification of DSVI as ‘’true’’ and ‘’missed’’ DSVI. Missed-DSVI was defined as having no SVI seen in the initial CECT arterial phase due to the late arterial phase acquisition that probably failed to show a potential SVI. (Fig. 3).

### 2.5 Statistical analysis

Cases were presented as absolute numbers and percentage. Continuous variables were expressed by mean and standard deviation (SD), Table 2. Association between continuous variables were studied using Pearson correlation for non-normal data. Association between categorical variables were assessed by fisher exact test (for any observation < 5), Cochran-mantel-Haenszel test, and Chi-square test as appropriate. Logistic regression was used to analyze the relationship between NOM outcome (success/failure) as dependent qualitative variable and other independent variables: age, gender, mechanism of injury, ICU length of stay, and initial CTSI score. Analyses were performed using Statistical Analysis Software (SAS) Enterprise Guide© version 8.1 and Statistical Package for the Social Sciences (SPSS) 64-bit version 25.

**Table 2:** Summary of cases characteristics.

Variable	Overall
N	99
Gender = MALE (%)	92 (92.9)
Age (mean (SD))	32.69 (12.31)
Mechanism of Injury (%)	
Assault	3 (3.0)
Blunt trauma	4 (4.0)
Fall	26 (26.3)
Road traffic accident	66 (66.7)
Mortality (mean (SD))	0 (0.0)
TICU stay (mean (SD))	6.6 (5.3)
Initial Management (%)	
Conservative	50 (50.5)
Angiogram	49 (49.5)
Initial CTSI (%)	
1	7 (7.1)
2	28 (28.3)
3	28 (28.3)
4a	31 (31.3)
4b	5 (5.1)
Embolization Technique (%)	
Combined	10 (22.2)
Distal	11 (24.4)
Proximal	24 (53.3)

## 3 Results

### 3.1 Descriptive statistics (Table 2)

#### 3.1.1 Study population: baseline data

From January 2013 to August 2021, 129 patients presented to emergency department with blunt splenic trauma. The analyses were conducted on the remaining (99/129) patients who fulfilled the inclusion criteria. The most common causes of splenic injury were road traffic accident (RTA) (66/99), falling from height (26/99) and other causes (7/99). (92/99) subjects were males. The average sample age was 32.7 ± 12.3 years old. The mean TICU stay was 6.6 ± 5.3 days (Table 2).

#### 3.1.2 CTSI Grading and management

A total of (63/99) patients had SPI without SVI: (35/63) patients found to have low-grade injury (7/35 CTSI grade 1 and 28/35 scored CTSI grade 2); (28/63) patients had intermediate-grade injury and scored CTSI grade 3; and (36/99) patients had high-grade injury of which 31 cases scored CTSI grade 4a. Among the latter category: (24/31) patients had pseudoaneurysms; (2/31) had intraparenchymal contrast extravasation; (1/31) patient had shattered spleen associated to intraparenchymal extravasation and (4/31) patients had shattered spleen without SVI. Active intra-peritoneal contrast extravasations were observed in (5/36) patients and scored CTSI grade 4b.

A total of (49/99) patients underwent splenic angiography: (32/49) patients had initial SAE; (13/49) patients had initial CM but subsequently needed SAE due to DSVI; while (4/49) patients were planned for SAE but only went through angiography without embolization as SVI could not be detected in angiography.

The concordance of the angiogram findings and CT features of severe parenchymal injury or SVI prior to the angiogram was noted in (45/49; 91.8%) patients. (4/49; 8.2%) patients had features of SVI in CECT but no discernible SVI features in the angiogram study afterwards; possibly to thrombosis of the pseudoaneurysm after the examination (false negative). 24/45 patients who had SAE underwent proximal SAE, (11/45) had distal SAE, and (10/45) had combined proximal and distal SAEs. (4/45) patients had to undergo another SAE due to complex splenic vascular anatomy making the first SAE difficult to realize. (50/99) patients underwent CM, of which (3/50; 6%) patients needed secondary splenectomy due to hemodynamic instability, and (3/50; 6%) patients were found to have DSVI managed conservatively.

#### 3.1.3 Success and failure rates

Overall, (90/99; 90.9%) patients had successful NOM, specifically: (47/50) after CM and (43/49) after SAE. Among 9/99 patients who had clinical failure. The initial CTSI grading of the clinical failure group; grade 3 (2/9), grade 4a(4/9), and grade 4b (3/9). The mortality rate of both clinical success and failure patients was (0%).

#### 3.1.4 DSVI

15/99 (15.2%) patients had DSVI in follow up CT. After reviewing the initial CECT, (8/15) were found to have ‘’true’’ DSVI after initial NOM. Out of these (5/8) needed SAE and (3/8) had CM. (7/15) were considered as ‘’missed’’ DSVI as the SVI in this group was overlooked in the initial CECT, presumably due to late arterial phase acquisition. Cases with initial CTSI score of 3 found to be statistically significantly associated with DSVI on follow up CT (P < 0.05).

### 3.2 Statistical analysis

TICU (in days) were weakly correlated with severity of CTSI [Pearson correlation coefficient 0.2, p-value 0.04]. There is a statistically significant association between CTSI grade 3 injury and the development of DSVI (p < 0.05). There is an association between severity of initial CTSI score and the risk of NOM/clinical failure [p-value = 0.02]. After adjusting for age, gender, mechanism of injury, and TICU length of stay, CTSI remains the main predictor of failure of NOM [p-value 0.01].

## 4 Discussion

Numerous scoring systems have been developed to categorize the severity of splenic injury, aiming to standardize delivered management and predict outcomes of patients with blunt splenic injury [13]. The use of the radiologic scoring system was achievable through the development of the AAST and CTSI scoring systems [11], [12]. Both systems provide an anatomical description of SPI in addition to providing valuable information concerning associated SVI, which helps in standardized therapeutic decisions with regard to either CM or SAE. It has been reported earlier that CTSI provides better prediction of NOM outcomes than AAST-1994 [3], [11], [14]. Thus, our institution decided to implement the CTSI scoring system instead of AAST-1994 to plan for NOM. Our decision was endorsed later by evidence showing similar outcomes of CTSI to the newly modified AAST scoring system (AAST-2018) in characterizing splenic injury grade.

Furthermore, it has been demonstrated that CTSI is superior to AAST-2018 in predicting mortality [10]. Therefore, CM was reserved for CTSI grades 1 and 2, while SAE was indicated in the presence of high-grade SPI and/or evidence of SVI (CTSI grades 4a and b) [15]. CTSI grade 3, however, is a grey area for which the indication for either CM or SAE is controversial [16]. In our institution, an adjunctive decision was taken with the trauma team to manage CTSI grade 3 patients conservatively for 72 h, followed by CECT before either patient discharge or decision for reintervention.

Our study included ninety-nine blunt splenic injuries with a success rate of NOM reaching 90.9%, which is consistent with the results of other studies [17], [18], [19], [20], [21], [22]. As expected, failure was observed more in patients who had a high-grade injury (grades 4a and b) [23]. The main cause of failure in this group was the technical failure to achieve proper embolization leading to splenectomy. While some authors defined failure of NOM as failure to conserve the spleen after an initial NOM, others expanded this definition by adding SAE to splenectomy in case of failure of initial NOM [24].

SAE was performed on (45/99) patients. Regardless of the technique, SAE was clinically successful in achieving hemostasis in (44/45; 98%) patients, which is in line with the results of other studies showing a success rate of 96–98% [17], [18], [19], [20], [21], [22].

Although the indication for SAE was based on CTSI grading (grade 4a and b), the embolization technique (proximal, distal, or combined) was planned based on the severity of SPI and on the site and number of SVI detected in the CECT. The plan was then adjusted depending on the angiogram findings. In the early phase of our study, coils were our preferred embolic agents for proximal SAE. Due to incidences of dislodgement, we decided to replace coils with Amplatzer Vascular Plugs (AVP), which did not demonstrate any further material migration. Regarding distal embolization, coils were commonly used.

Different factors were suggested to be associated with clinical failure in the literature. These include patient’s age, associated solid organ injuries, and the amounts of hemoperitoneum [16], [25], [26], [27]. Many of these parameters are not included in the CTSI grading systems and have not been taken into consideration in our study [2], [28], [29], [30]. 15 (15.2%) patients had DSVI on follow-up CECT after an initial NOM plan. DSVIs were present in CTSI grade 2 (2 patients) and in CTSI grade 3 (13 patients). This demonstrates the effectiveness of SAE in managing high-grade lesions (grades 4a and b). Twelve patients with DSVI underwent SAE, and three had CM. None of our patients with DSVI needed surgical splenectomy. Our results showed a statistically significant association between CTSI grade 3 injury and the development of DSVI (p < 0.05). This raises interrogations regarding the efficiency of CM in patients with CTSI grade 3. Many reports have discussed the causes of DSVI after NOM. Thus, Jahromi et al. attributed splenic rebleeding after an initial NOM to a subcapsular hematoma that was not detected by CECT early after injury but subsequently expanded and ruptured [31]. Furthermore, Uyeda et al. Escalated the importance of dual-phase CT protocol in detecting arterial SVI in the initial CECT [32]. Further, Leepr et al. pointed to the subsequent rupture of splenic pseudoaneurysms to explain DVSI, which remains the rationale for CT follow-up [33].

DSVI has been variably reported to range between 3%− 15% with unclear association with splenic injury severity [33], [34], [35], [36], [37]. Since (7/18; 25%) of CTSI grade 3 patients were associated with true DSVI, which is considerably higher than grade 1 or 2, we suggest extending the follow up period for CTSI grade 3 up to 14 days. Further, the review of the CECT of our cases showed that 7/15 patients were considered as ‘’missed’’ DSVI. The SVI was not detected in the initial CECT due to late arterial phase acquisition, at which the SVI may appear isodense to splenic parenchyma. Our study did not find any subcapsular hematoma contributing to delayed bleeding.

As no consensus exists regarding follow-up CECT practices in the literature, the issue of repeating CT, especially in CTSI grade 3 splenic injuries, needs further investigation. Selective repeated imaging aims to identify delayed or latent vascular anomalies and address them with embolization that would improve overall splenic salvage. Davis et al. suggested a repeated CT protocol for inpatients within 48 h of initial abdominal CT [34]. They reported that half of the detected delayed splenic vascular abnormalities were not found in the initial CT scan of the spleen, and therefore advocating for repeating CT imaging. Savage et al. reported that 80% of splenic injuries of grade 1 and 2 showed complete healing of the spleen at post-injury day 50, suggesting that repeating CT is not worthwhile in low-grade injury unless the patient develops signs and symptoms suggestive of intra-abdominal hemorrhage [38]. On the other hand, 10% of patients with high-grade splenic injury had worsening of their injuries in repeated images, and only two patients needed splenectomies. In our institution, CECT is repeated at 72 h of admission for all patients with splenic injuries Grade 3 and above. For splenic injury in grades 1 and 2, the CECT is repeated only in case of deterioration of hemodynamic status during the admission after initial NOM suspecting DSVI.

The CTSI scoring system showed some limitations: first, CTSI grades 4a and 4b incorporate different grades of SPI with or without SVI. For each type, a different technique of SAE is selected. Thus, patients with high-grade SPI will have proximal or combined SAE based on the presence or not of a concomitant focal SVI. On the other hand, patients with low-grade SPI require either distal or proximal SAE based on the presence of focal or diffuse SVI. Even if there is no firm evidence of the superiority of each SAE technique over the other, we believe that standardization of the technique of SAE based on the grade of SPI and the presence of SVI or not will have a better impact in terms of clinical management and outcome. Second, according to EAST study, it has been shown that the amount of hemoperitoneum may reflect the severity of the splenic injury, and it can be predictive of NOM failure [2], [15], [39]. However, hemoperitoneum is not taken into consideration by the CTSI scoring system. Third, following CTSI criteria, any vascular injury will upgrade the severity of injury to grade 4a or b. However, the majority of small pseudoaneurysms (less than 10 mm) and patchy focal patterns in the initial CECT will spontaneously thrombose without intervention [40]. Upgrading these patients to grade 4 may indicate unnecessary SAE.

Our study represents one of the largest sample size single-center experiences on managing blunt splenic trauma using the CTSI scoring system over a long study period to be reported in the Middle East region. This study included comprehensive data on injury patterns, NOM details, and outcome parameters; therefore, we believe it can add to the available knowledge in this specific subject.

The study had several limitations. First, due to the study's retrospective design, we might have missed some clinical data that may add further information. Second, our study did not investigate patient age and hemoperitoneum degree since we were targeting the parameters incorporated in the CTSI scoring system only. Third, despite respecting the triphasic protocol of CECT in our study, in some cases, the early arterial acquisition was not taken into consideration, which led to misinterpretation of the initial CECT. Fourth, due to the long duration of the study, CECT interpretation had many variations of splenic injury, mainly in the early phase of the study. This variation has been reduced by assigning two dedicated emergency radiologists to review all initial CECTs and adjust the variation. Finally, many patients did not show up during the follow-up; consequently, some cases of DSVI after discharge could not be reassessed.

## 5 Conclusion

Our study confirms the role of CTSI scoring system as a practical tool in predicting the type of NOM and outcomes of patients with blunt splenic trauma. According to our findings, implementing such a system in a level 1 trauma center will further improve the outcome of treatment for splenic blunt trauma. However, CTSI grade 3 is considered an increased risk of NOM failure, and further investigations are necessary to standardize its management.

## Ethical approval

For this type of study formal consent is not required. The study protocol was approved by the institutional review board (MRC-01–21–294).

## Informed consent

For this type of study informed consent is not required.

## Consent for publication

For this type of study consent for publication is not required.

## Funding

This research did not receive any specific grant from funding agencies in the public, commercial, or not-for-profit sectors.

## CRediT authorship contribution statement

**Kassamali Rahil:** Writing – review & editing, Writing – original draft, Supervision. **Al Hasani Ammar:** Writing – review & editing, Writing – original draft, Supervision. **Barah Ali:** Supervision, Methodology, Investigation, Formal analysis, Data curation, Conceptualization. **Al-Thani Hassan:** Writing – review & editing, Writing – original draft, Supervision. **Omar Ahmed:** Writing – review & editing, Writing – original draft, Supervision. **M AlAhmad Yaman:** Writing – review & editing, Writing – original draft, Investigation, Formal analysis. **Jaleel Zeyad:** Writing – review & editing, Writing – original draft, Supervision. **Elmagdoub Ayman:** Supervision, Methodology, Investigation, Formal analysis, Data curation, Conceptualization. **Aker Loai:** Supervision, Methodology, Investigation, Formal analysis, Data curation, Conceptualization. **Ahmed Zahoor:** Writing – review & editing, Writing – original draft, Supervision.

## Declaration of Competing Interest

The authors declare that they have no known competing financial interests or personal relationships that could have appeared to influence the work reported in this paper.
